# Case Report: Bimekizumab for dissecting cellulitis of the scalp

**DOI:** 10.3389/fimmu.2026.1830178

**Published:** 2026-05-19

**Authors:** David Seiter

**Affiliations:** Epiphany Dermatology, Phoenix, AZ, United States

**Keywords:** acne keloidalis nuchae, bimekizumab, dissecting cellulitis of the scalp, follicular occlusion tetrad, IL-17 A/F inhibitor

## Abstract

Dissecting cellulitis of the scalp (DCS) is a cicatricial alopecia characterized by follicular occlusion that progresses to abscess formation and rupture, driven by an intense inflammatory cascade. Part of the follicular occlusion tetrad, DCS typically presents with painful nodules and interconnecting sinus tracts localized to the scalp. Treatment of this recalcitrant disease is difficult, and there is a need for efficacious and durable therapies that control inflammation and address the underlying pathophysiology of disease. Here is the first reported use of bimekizumab, a dual inhibitor of interleukin (IL)-17A and IL-17F, as monotherapy for the management of DCS. The patient was a male aged 27 years who presented with difficult-to-treat DCS and hair loss. After failing to achieve symptomatic control with standard treatments such as oral antibiotics, aggressive topical therapy, and intralesional steroids, the patient received bimekizumab (320 mg subcutaneously every 2 weeks through week 16, and 320 mg every 4 weeks thereafter). At the 16-week follow-up, the patient reported no symptoms of DCS or adverse events from treatment. The outcomes from our case report provide evidence supporting the inhibition of IL-17A and IL-17F for the treatment of DCS to address the underlying inflammatory mechanism, reduce symptoms, and help patients achieve control over their disease and improve their overall quality of life.

## Introduction

Dissecting cellulitis of the scalp (DCS), a component of the follicular occlusion tetrad (FOT), often presents with painful nodules, abscesses, and interconnecting sinus tracts localized to the vertex and occipital regions of the scalp (1, 2). DCS primarily affects men between the ages of 18–40 years and is most prevalent in African American men (3, 4). Onset of DCS may occur between 11 and 50 years of age (4). Diagnosis of DCS is clinical; however, histopathology may also aid in diagnosis and determining stage and severity of disease (1). DCS is progressive and over time can lead to disfigurement of the scalp and permanent hair loss (1). Given the underlying inflammatory burden and similar presentation, some have described DCS as a localized variant of hidradenitis suppurativa (HS), another component of the FOT (4, 5). Patients with DCS, like those with HS, report poor quality of life and experience anxiety, depression, and social isolation (3, 6). Further, the chronic, relapsing nature of DCS makes it difficult to manage (2). No standardized, evidence-based guidelines for DCS exist, and treatment approaches are often drawn from case reports (5). Treatment involves topical and systemic antibiotics along with intralesional or oral corticosteroids to reduce inflammation (1, 7). Oral retinoids are also used to treat DCS (7), and incision and drainage may provide acute relief. Procedural therapies, including laser and excision, have also been employed in severe cases (7). While these treatments provide temporary relief, the underlying cause of inflammation often remains unaddressed, which can lead to disease recurrence (1, 5, 8). Drawing on their understanding of the shared pathology between DCS and HS, clinicians have reported case studies on the treatment of DCS with biologics that have shown efficacy in patients with HS (8). Masson et al. reviewed the use of biologics to reduce the inflammatory load in patients with DCS and found that some patients experienced complete lesion clearance, while others failed to adequately respond or experienced symptom relapse (7). There are also reports of using combinations of therapeutic approaches for patients with DCS, suggesting that reliable symptom control is difficult to achieve (7). There is a need for efficacious, tolerable, and durable management options for DCS that address the underlying pathophysiology and resulting symptoms of the disease. Here is the first reported use of bimekizumab, a humanized monoclonal antibody that selectively inhibits interleukin (IL)-17A and IL-17F (approved for the treatment of HS and other inflammatory conditions), as monotherapy for the management of difficult-to-treat DCS (9).

## Case report

### Patient presentation

In May 2020, a Hispanic male aged 22 years who was a nonsmoker with an unremarkable medical history first presented with moderate focal hair loss on the scalp that had been present for months. Physical examination revealed discrete patches of hair loss and xerotic desquamating erythematous papules in some areas. The patient was diagnosed with alopecia areata and prescribed clobetasol 0.05% scalp solution.

At follow-up in January 2021, the patient transferred to our care and reported recurrent abscess-like lesions on the scalp that were enlarging, draining, tender, moderate in severity, and associated with hair loss. The patient clarified that he had experienced similarly inflamed, draining lesions prior to his May 2020 visit, though such lesions were not present at the time of initial presentation. Physical examination at the follow-up visit showed suppurative boggy plaques distributed on the midoccipital, right central parietal, posterior midparietal, and left central scalp. These findings, coupled with the patient’s history, prompted us to change the diagnosis to DCS; a biopsy was not obtained for histopathological confirmation.

### Clinical course

The patient was prescribed doxycycline hyclate 100 mg twice daily and sulfacetamide/sulfur 9.8%–4.8% topical cleanser to use to wash his scalp once daily. At follow-up, the patient reported some reduction in number, severity, and frequency of inflammatory lesions and associated hair loss. However, a physical examination showed persistent suppurative boggy plaques prompting treatment of these lesions with intralesional steroids (3 treatments of intralesional triamcinolone [5.0 mg/mL]) in addition to prescribed systemic antibiotics and topical cleanser.

At subsequent follow-up visits, the patient presented with pustules and keloidal papules on the posterior scalp resulting in the diagnosis of acne keloidalis nuchae (AKN) associated with DCS. The patient was prescribed doxycycline 50 mg twice daily for 3 months and continued use of sulfacetamide/sulfur 9.8%–4.8% cleanser daily.

In May 2023, at age 25 years, the patient returned to his previous provider reporting pruritic, alopecic lesions that ruptured and wept spontaneously. He stated that he did not adhere to the treatment plan for AKN or DCS as directed. The patient also reported a widespread breakout after shaving his scalp. The patient was prescribed minocycline 100 mg twice daily and over-the- counter benzoyl peroxide cleanser (2.5%–5%) daily, and he was encouraged to keep his hair no shorter than 1–2 cm in length.

**Figure 1:**
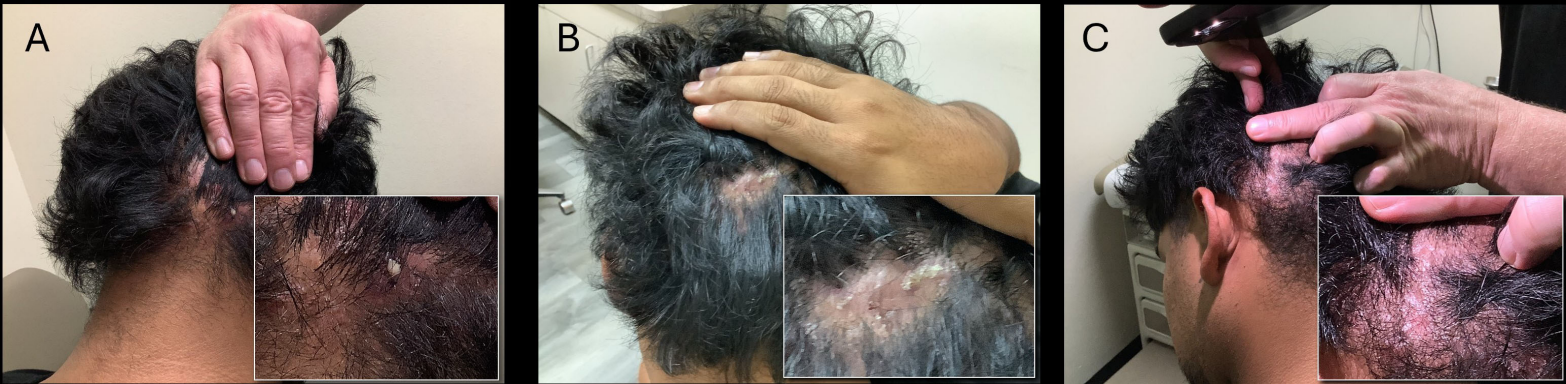
DCS with boggy suppurative patches and signs of hair loss on the **(A)** right posterior, **(B)** midoccipital, and **(C)** left posterior scalp prior to initiation of bimekizumab.

When the patient returned to our care in September 2025, at age 27 years, he continued to experience draining and bleeding alopecic lesions that were pruritic and painful (**Figure 1**). He noted that he was not adhering to the treatment plan of minocycline and benzoyl peroxide cleanser (2.5%–5%) but using doxycycline obtained from Mexico. The patient described his condition as uncomfortable and painful. His quality of life decreased across many areas: The itch and pain caused difficulties falling asleep; social interactions caused embarrassment due to weeping and blood stains present on his clothing, car headrest, and paper liner at the chiropractor; wearing hats was painful; and he had learned to cut his own hair, as he was too self-conscious to visit a barber. His personal relationships suffered, and he did not want his partner to touch his hair. He stated that his therapeutic journey had been a discouraging cycle of intermittent treatments, none of which provided satisfactory relief.

Review of the patient’s treatment history showed negligible improvement from topical therapies. Complete resolution was achieved while on two 30-day courses of doxycycline, and 75%–80% improvement was achieved while on a 30-day course of minocycline; however, the condition would flare and return to baseline severity between rounds of oral antibiotics. The patient did not respond to intralesional steroids.

**Figure 2:**
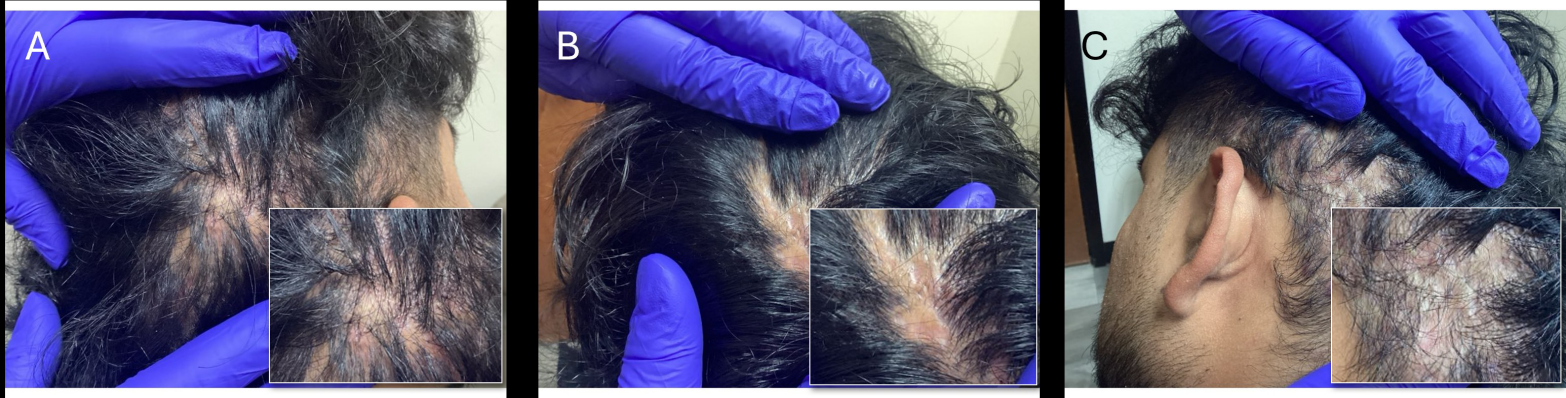
Follow-up visit at week 16 of bimekizumab treatment. The patient was clear of inflammatory lesions on the **(A)** right posterior, **(B)** midoccipital, and **(C)** left posterior scalp and there was evidence of hair regrowth.

**Figure 3:**
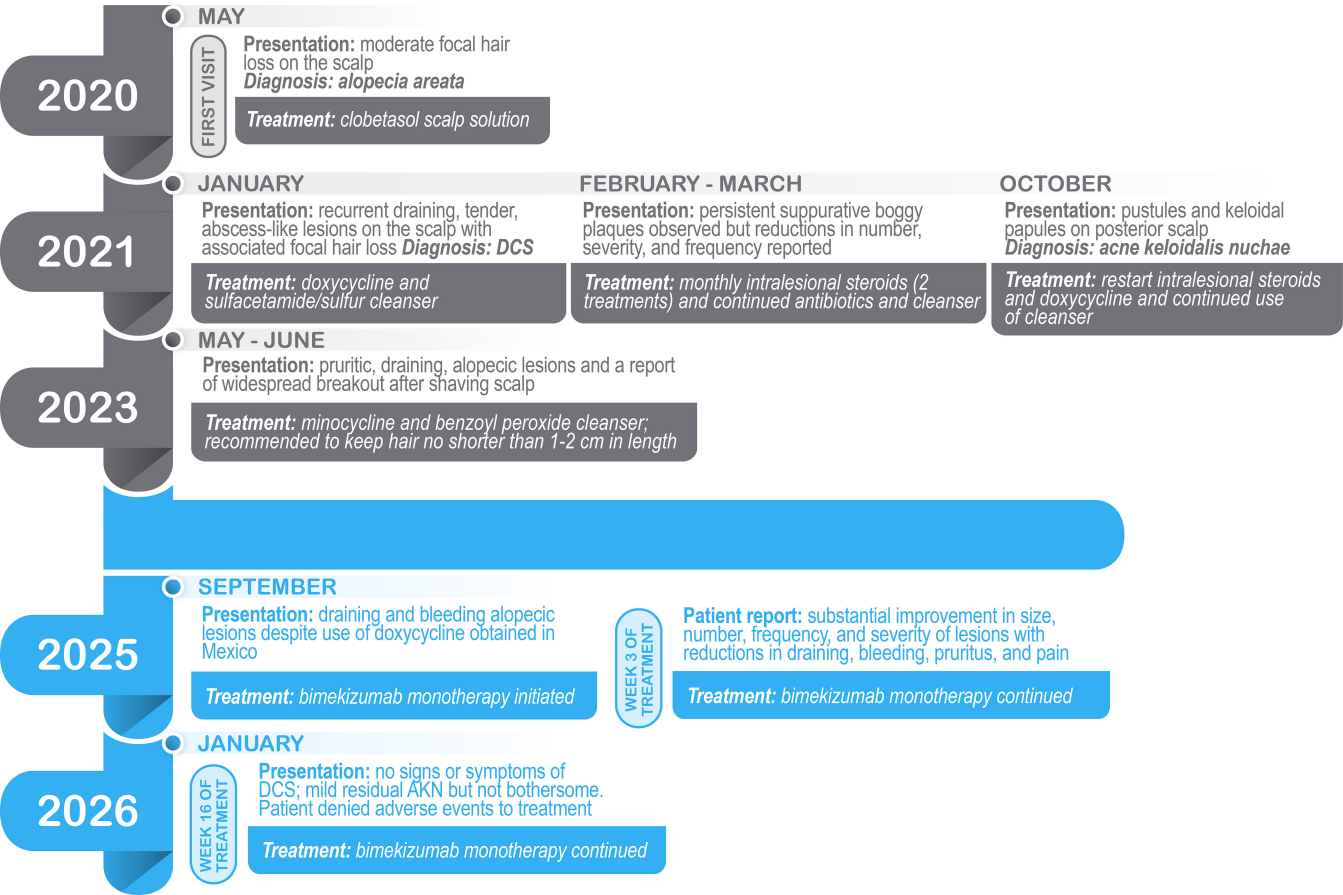
Patient symptom presentation, diagnosis, and treatment timline.

The decision was made to address his DCS with a biologic, and bimekizumab was prescribed (320 mg subcutaneously every 2 weeks through week 16, then 320 mg every 4 weeks thereafter). Laboratory tests (a complete blood count with differential and comprehensive metabolic panel as well as hepatitis B/C and tuberculosis screening) were ordered to determine eligibility for immunomodulatory treatment and ongoing monitoring. Three weeks after starting bimekizumab, the patient reported substantial improvement in size, number, frequency, and severity of inflammatory lesions and reductions in draining, bleeding, pruritus, and pain. At 16 weeks, the patient reported no symptoms of DCS and denied adverse effects of therapy. He also reported improvement in his quality of life. Physical examination showed that the DCS was well controlled with mild residual AKN on the posterior inferior scalp that was not bothersome. There were no inflammatory lesions, and the patient experienced approximately 70% hair regrowth (**Figure 2**). A timeline of the patient’s presentation, diagnosis, and treatment from first visit through week 16 of bimekizumab is summarized in **Figure 3**.

## Discussion

DCS is difficult to effectively manage due to its chronic and relapsing nature. Traditional therapies such as antibiotics may provide temporary relief (1). However, to control inflammation and minimize recurrence, prolonged use is often required (1), which increases the risk of antibiotic resistance and negatively alters patient microbiota (10). Some patients may experience durable symptom improvement from oral retinoids; however, their efficacy is often limited to mild disease (1, 7).

The pathobiology of DCS, like the other diseases of the FOT, is thought to involve dysregulation of inflammatory signaling pathways, notably including IL-1β, IL-17, tumor necrosis factor-alpha (TNFα), and the Janus kinase/signal transducer and activator of transcription (JAK/STAT) proinflammatory mediators (1, 5). Inhibition of IL-17 and TNFα have shown efficacy in treating HS and other inflammatory diseases (11, 12). Based on the potential shared pathobiology, it was hypothesized that DCS may also be managed by inhibiting the same inflammatory pathways. Cases of DCS treated with biologics (anti-TNFα, anti-IL-23, anti-IL-17A, and JAK/STAT inhibitors) have been reported, with some therapies demonstrating more effectiveness than others (7, 11). An understanding of the aberrant signaling pathways and predominant inflammatory mediators in DCS may give insight into which biologics may be most effective in treating this complex disease.

Activation of the innate immune system including production of TNFα and IL-17A/F inflammatory mediators may be assumed to be key drivers of the nodules, abscesses, and other manifestations characteristic of DCS (5). In many autoimmune diseases, IL-17A/F production is largely regulated by IL-23 (13); however, Cole et al. demonstrated IL-23 independent production of IL-17A/F by mucosal-associated invariant T cells (14). Mucosal-associated invariant T cells may be activated by innate cytokines or by vitamin B metabolites (signs of bacterial infection) presented by major histocompatibility complex–related protein 1 to produce IL-17, TNFα, and interferon-ɣ (15). Further, Rastrick et al. found that mucosal-associated invariant T cells are highly concentrated in HS lesions and produce predominantly the IL-17F isoform (16). These findings may explain why patients with HS respond well to IL-17A/F inhibition and less favorably to IL-23 inhibition (5, 9, 17). This is why an IL-17A/F inhibitor was selected rather than one of the other available biologics for our patient. As predicted, the patient experienced rapid improvement in his DCS symptoms following initiation of bimekizumab and achieved complete disease control by week 16.

During the patient’s journey of managing his DCS, he presented with an associated case of AKN. AKN is a cicatricial alopecia caused by dysregulated inflammation of unknown etiology (18, 19). Interestingly, while the patient was being treated with bimekizumab, there was a reduction in AKN-associated pruritus, and no new lesions formed, suggesting potential involvement of IL-17A/F in AKN pathobiology as well. Caudrelier et al. presented a unique case showing a patient who was prescribed upadacitinib for atopic dermatitis and showed symptom improvement of a concurrent case of AKN, suggesting potential involvement of JAK/STAT signaling in AKN (18). Based on these observations, it may be likely that the deep reduction of inflammation through IL-17A/F inhibition also attenuates the localized inflammatory signaling in AKN. Further studies investigating the possible link between DCS and AKN are needed to fully understand any shared pathobiology.

This case report is limited by describing results of only one patient with DCS treated with BKZ as monotherapy. Additional studies are required to confirm the efficacy of bimekizumab in this population. Further, while our patient experienced improvements in symptom control and quality of life through week 16, longer-term observations are required to assess the durability of response to bimekizumab in DCS.

## Conclusions

This report describes what is believed to be the first reported use of bimekizumab as monotherapy for the treatment of difficult-to-treat DCS. With a mechanistic understanding of the pathobiology of the FOT and the key cytokines involved in HS, it was theorized that inhibition of IL-17A/F would provide relief to the patient, especially after he was not able to find a deep and durable response with first-line treatment options. Treatment with bimekizumab alone resulted in significant improvement in the size, number, frequency, and severity of our patient’s inflammatory lesions. During the 16-week follow-up, no inflammatory lesions were found on physical examination; the patient reported no symptoms of DCS, and the patient experienced hair regrowth in many areas of hair loss. While this is only a single patient case of DCS being treated with bimekizumab, the outcomes emphasize the importance of treating the underlying inflammatory condition to achieve durable symptom relief, especially as these results were achieved with bimekizumab alone, and not in combination with other therapies. Further research is needed to understand the effect of inhibiting IL-17A/F and other inflammatory cytokines in the treatment of DCS. The success of this case represents an opportunity to improve our management of DCS and advance our understanding of the inflammatory mechanisms underlying DCS and associated diseases.

## Data Availability

The original contributions presented in the study are included in the article/supplementary material. Further inquiries can be directed to the corresponding author.
